# Development and piloting of a food‐based intervention to increase vitamin E intake in pregnant women in a randomized controlled trial

**DOI:** 10.1002/fsn3.353

**Published:** 2016-03-02

**Authors:** Julia Clark, Nikki Holgan, Leone Craig, Heather Morgan, Peter Danielian, Graham Devereux

**Affiliations:** ^1^ Public Health Nutrition Research GroupUniversity of AberdeenAberdeenUnited Kingdom; ^2^ New Product Development TechnologistBaxters Food GroupFochabersUnited Kingdom; ^3^ Public Health Nutrition Research GroupUniversity of AberdeenAberdeenUnited Kingdom; ^4^ Health Services Research UnitUniversity of AberdeenAberdeenUnited Kingdom; ^5^ ObstetricianAberdeen Maternity HospitalAberdeenUnited Kingdom; ^6^ University of AberdeenAberdeenUnited Kingdom

**Keywords:** Asthma, food, pilot randomized controlled trial, pregnancy, Vitamin E

## Abstract

Low maternal vitamin E intake during pregnancy is associated with childhood asthma and a trial is required to test whether increasing maternal vitamin E intake reduces childhood asthma. This study investigated whether such a trial is possible using food to increase vitamin E intake. Three soup varieties with enhanced vitamin E content (16–19 mg/can) from food ingredients were developed. Near identical retail versions (vitamin E 1–4 mg/can) acted as placebo. In a pilot double‐blind randomized controlled trial, pregnant women were randomized 1:1 to enhanced or placebo soups (three tins/week) from 12 weeks gestation to delivery. Vitamin E intake was quantified at 12, 20, and 34 weeks gestation. Qualitative interviews were conducted. 59 women were randomized (29 enhanced, 30 placebo), 28 completed the trial, (15 enhanced, 13 placebo). In women completing the trial, vitamin E intake of the placebo group remained unchanged; 7.09 mg/d (95% CI 5.41–8.77) at 12 weeks, 6.41 mg/d (5.07–7.75) at 20 weeks, and 6.67 mg/d (5.38–7.96) at 34 weeks gestation; vitamin E intake of the enhanced group increased from 6.50 mg/d (5.21–7.79) at 12 weeks to 14.9 mg/d (13.3–16.4) at 20 weeks and 15.2 mg/d (12.9–17.5) at 34 weeks, *P *< 0.001. Qualitative interviewing provided clear guidance on improving adherence. Although 31 women withdrew at median 19 weeks gestation (interquartile range 16–25), the intervention was consumed by women for 80% of weeks between 12 and 34 weeks gestation and for 63% of weeks between 12 weeks gestation and delivery. In a pilot double‐blind randomized controlled trial (RCT) it is possible to increase maternal vitamin E intake using food ingredients, a further food product is required to improve adherence.

## Introduction

It has been hypothesized that changes in maternal diet during pregnancy contributed to the recent increase in asthma (Devereux 2006). In observational cohort studies associations have been reported between low maternal dietary vitamin E intake during pregnancy and increased risk of childhood asthma (Nurmatov et al. 2011; Allan et al. 2015), and trials of vitamin E intervention during pregnancy for the primary prevention of childhood asthma have been recommended (Nurmatov et al. 2011; Allan et al. 2015). Given that we do not consume single nutrients or foods in isolation and some nutrients may act synergistically to exert a beneficial effect, an intervention based on vitamin E containing foods is more physiological and, moreover, avoids issues associated with pharmaceutical supplementation (Lawlor 2004). In the UK the vitamin E intake of women of childbearing age has been reported to be about 8 mg/d (Henderson et al. 2003; Devereux et al. 2006) which is somewhat less than 15 mg/d, a level that has been recommended for pregnant women (Institute of Medicine; Food and Nutrition Board, 2000).

In this short report, we describe the development and piloting of a novel food‐based intervention suitable for a randomized controlled trial (RCT) of increasing maternal vitamin E intake to 15 mg/day in pregnant women using food ingredients. The acceptability and effects on vitamin E intake of piloting the intervention throughout pregnancy in a double‐blind placebo‐controlled RCT are also presented.

## Method

### Intervention

The intervention was designed on a baseline vitamin E intake of 8 mg/d, the value reported for UK women of childbearing age (Henderson et al. 2003; Devereux et al. 2006). In collaboration with a locally well‐known food producer (Baxters Food Group), three soup varieties were developed based on the popular varieties; tomato, puy lentil & tomato and bean & pasta. The soups were designed around the availability of ingredients naturally rich in vitamin E that the food manufacturer's suppliers could provide and the limitations imposed by the factory being nut‐free, for example, sun‐dried tomatoes, sunflower oil, rapeseed oil, wheatgerm, and butternut squash. Vitamin E supplements were not added. The costs of the soups were kept to a minimum by avoiding meat sources, thus allowing the intervention to be suitable for vegetarians. Each tin contained 16–19 mg vitamin E so that consumption of three tins a week would increase mean vitamin E intake to 15 mg/d. Retail versions of the soups were used as placebo. This also meant that the retail soups could easily be used at family meal‐times while the participant had the intervention soup, thus allowing for easier meal planning. The soups were designed to replace a meal and participants were also encouraged to take the product to work with them by providing a fashionable lunch‐bag. Several rounds of taste testing of the intervention soups took place to ensure palatability and similarity to retail versions. Food tables were used to predict vitamin E content but actual content was confirmed by HPLC. The *α*‐Tocopherol content/tin of enhanced soups was 16.2 mg (tomato), 19.0 mg (puy lentil) and 19.3 mg (bean & pasta) and for respective placebos 4.16 mg, 1.64 mg and 0.83 mg. Enhanced and placebo soups were packaged and labeled in a standardized fashion with enhanced/placebo status designated by a label code. To ensure double‐blinding the code was not released until after database lockdown.

### Pilot RCT

Healthy pregnant women with a personal or partner history of asthma attending Aberdeen Maternity Hospital at 12 weeks gestation were recruited. The aim was to recruit 50 women and randomize 25 to enhanced, and 25 to placebo intervention. A computerized randomization system allocated women to enhanced or placebo arms with equal probability. The trial was double‐blinded. Women were recruited and supported through pregnancy by a dietitian who also provided healthy eating advice as part of routine antenatal care.

Dietary intake at 12, 20, and 34 weeks gestation was assessed by a 4–day (3 weekday, 1 weekend day) nonweighted diet diary using photographic examples of food measures, the diary was analyzed using WISP (Tinuviel software, http://www.tinuvielsoftware.co.uk/wisp4.htm). Women were provided with free supplies of soups from 12 weeks gestation until delivery and asked to consume three cans a week during this period. Dietary vitamin E intake was analyzed using repeated measures ANOVA. Under‐reporting of energy intake at 12 weeks gestation was ascertained using the method of Goldberg et al. (Goldberg et al. 1991). Once the intervention was completed (delivery of child), women were invited to participate in one‐to‐one structured qualitative interviews conducted by different researchers. Interview transcriptions were entered into NVivo10 software (QSR International, Burlington, MA, USA) and independent analysis was informed by the Framework approach (Ritchie and Spencer 1994) until saturation of emerging themes. The trial was registered with www.clinicaltrials.gov (NCT01661530) and received ethical approval from the North of Scotland Research Ethics Service (12/NS/0053), participants provided written informed consent.

## Results

Invitation letters were sent to 661 women, 499 did not respond, 27 eligible respondents were not interested, 135 respondents were ineligible. In total, 59 women were recruited, mean age 31.6 years (95% CI 30.7–32.5), 29 were allocated to enhanced soup and 30 to placebo. 28 women completed the trial, 15 in the enhanced arm, 13 in the placebo.

**Figure 1:**
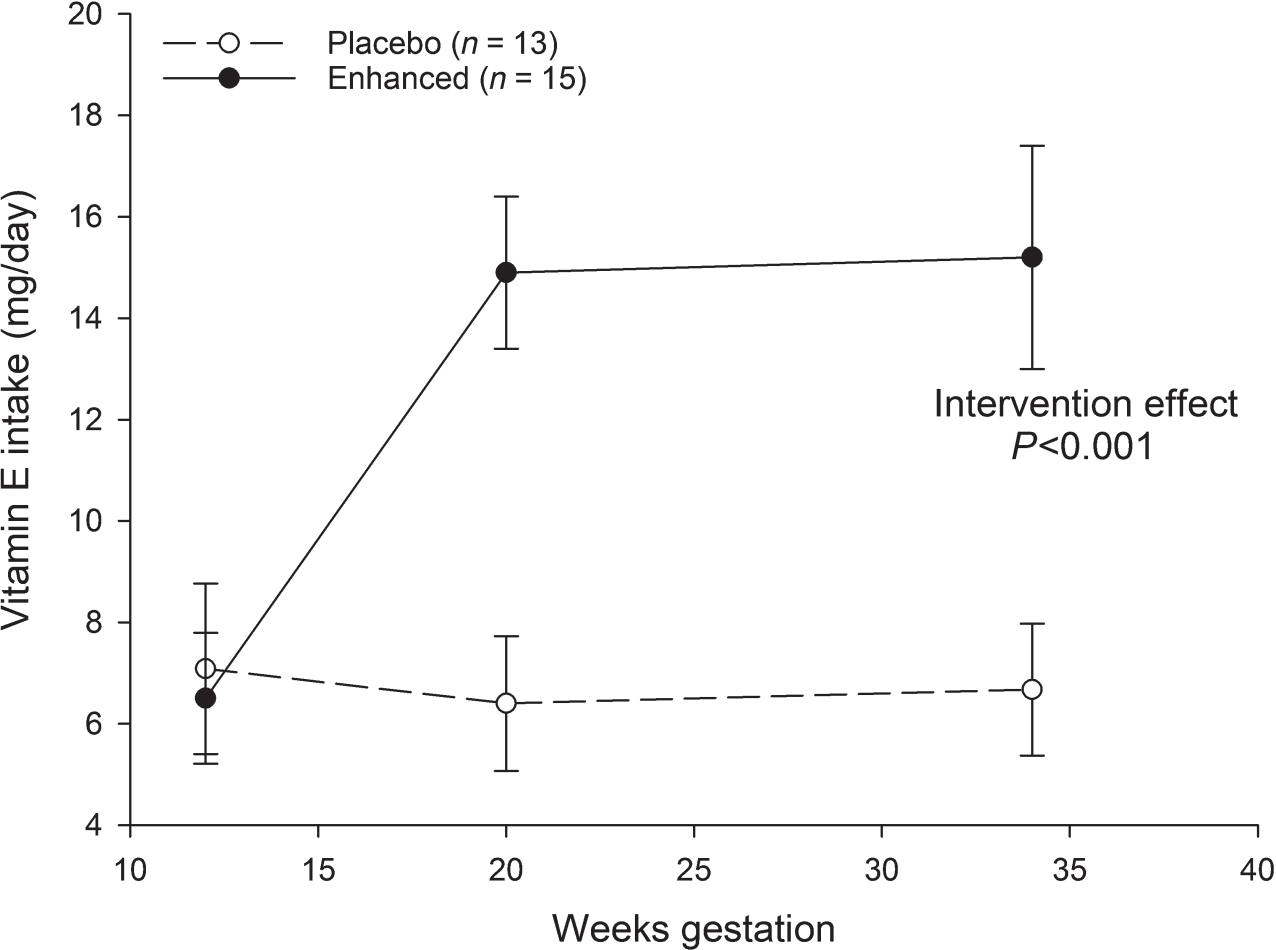
Food diary estimates (mean 95% CI) of vitamin E intake for women allocated to enhanced and placebo interventions.

Thirty one women withdrew at median 19 weeks gestation (interquartile range 16–25). Therapeutic coverage (the proportion of weeks for which the intervention was consumed) between 12 and 34 weeks gestation was 80%, and for the weeks between 12 weeks gestation and delivery therapeutic coverage was 63%. In women completing the trial, the dietary vitamin E intake of the placebo group (*n* = 13) remained unchanged being 7.09 mg/d (95% CI 5.41–8.77) at 12 weeks, 6.41 mg/d (5.07–7.75) at 20 weeks and 6.67 mg/d (5.38–7.96) at 34 weeks gestation. In women completing the trial, the dietary vitamin E intake of the enhanced group (*n* = 15) increased from 6.50 mg/d (5.21–7.79) at 12 weeks to 14.9 mg/d (13.3–16.4) at 20 weeks and 15.2 mg/d (12.9–17.5) at 34 weeks (Fig. 1). The intervention effect was significant, *P* < 0.001. There was no difference in mean energy intake at 12 weeks gestation between women allocated to placebo (1781 kCal/d, 95% CI 1556–2006) or enhanced soup (1773 kCal/d 95% CI 1586–1961). The intervention increased energy intake (~200 kCal/d, *P* = 0.023) and intakes of monounsaturated and polyunsaturated fatty acids (both *P* < 0.001). More women allocated to the placebo group under‐reported their energy intake at 12 weeks gestation than women allocated to the enhanced group (23% vs. 13%) although this was not statistically significant (*P* = 0.639, Fishers exact test). Restricting analysis to those women who did not under‐report energy intake reduced the difference in energy intake between enhanced and placebo group (~150 kCal/d, *P* = 0.078) but had negligible effect on the magnitude of the intervention effect for vitamin E, monounsaturated and polyunsaturated fatty acids (all *P* < 0.001). There were no statistically significant changes in intakes of protein, carbohydrate, saturated fat, cholesterol, sugars, fiber, zinc, selenium, retinol, beta‐carotene, vitamin D, and vitamin C.

The qualitative interviews of 47 women (25 enhanced, 22 placebo) revealed that most found consuming soup thrice a week was monotonous and too much, this was frequently spontaneously linked with suggesting a cereal bar alternative. Having the option of soups, bars or a combination was popular. A snack option was also popular. Consuming soups during summer was not so well‐liked, but many participants who already liked soup were keen during winter. The majority of women tended to consume ½–¾ tin at a time with the full contents of the tin being consumed over more than one sitting; there was minimal evidence of sharing with other family members; soups were considered ideal options when at work or time‐limited. The range and presentation of the soups was not considered a problem, although some women expressed concern about the absence of detailed nutritional information on the packaging, but acknowledged that such information may compromise the study.

## Discussion

Previously we have shown that maternal vitamin E intake during pregnancy can be increased to 15 mg/d using food exchanges (Clark et al. 2012), however, this was short term (12–20 weeks gestation), not standardized, required dietetic input and could not be blinded or placebo‐controlled. This study addressed these issues and demonstrated that in an RCT about half of pregnant women are willing and able to increase their vitamin E intake for the duration of pregnancy using food ingredients in the form of a food product (soup). This novel food‐based medium for increasing vitamin E meant that participants were not expected to take supplements which may have put some pregnant women off taking part. Also, the soups were designed to replace a meal, making them a convenient alternative for lunch, especially for those who worked. The qualitative post trial interviews confirmed the anticipated reasons for withdrawal, example somewhat monotonous nature of soup, but highlighted a possible solution to improve adherence, with a consensus that the intervention would be enhanced and made more acceptable by the development of cereal biscuits/bars to be used as a snack in warmer weather and to provide variety. There was no evidence that the intervention compromised macro/micro nutrient intakes.

The intervention was associated with increased energy intake, however, the magnitude of the increase (~200 kCal/d) was much greater than the expected 25 kCal/d increase based on the energy content of the enhanced soups. A sensitivity analysis indicated that an increased likelihood of under‐reporting of energy intake by the women allocated to the placebo group was the most likely explanation for the observed apparent increased energy intake with the intervention. The sensitivity analysis demonstrated that under‐reporting of energy intake had minimal impact on the magnitude of the intervention effect on vitamin E, monounsaturated and polyunsaturated fatty acid intake, possibly because of the relative ease of recording a can of soup in the food diary.

Although half of women completed the trial, therapeutic coverage was 63% of the weeks between 12 weeks gestation and delivery, suggesting that with a fairly modest increase in adherence the intervention will have the 80% adherence desirable for a RCT. Consequently, we are exploring the possibility of modifying retail cereal products to increase their vitamin E content using food ingredients example nuts, seeds, and oils. A further pilot study will be required to confirm improved adherence with a combined soup and cereal biscuit intervention. Our ultimate aim is to use the food‐based intervention in a multicentre double‐blind placebo‐controlled RCT to test whether increasing maternal dietary vitamin E intake during pregnancy reduces the risk of childhood asthma.

We did not measure serum tocopherol in this study because our previous pilot trial, of similar size, increasing maternal vitamin E intake during pregnancy to 15 mg/d using food exchanges was not associated with a detectable increase in serum tocopherol (Clark et al. 2012). A definitive large RCT of the proposed dietary intervention will, however, have the statistical power to detect the expected small increase in serum tocopherol. It will also be important to quantify the *α*‐ and *γ*‐stereoisomers of tocopherol given their differential effects on asthmatic airway inflammation in animal models (Cook‐Mills et al. 2013).

Although the focus of this study was vitamin E, the process of identifying nutrient rich food ingredients and developing food products containing specified quantities of nutrients for use as food‐based interventions could be transferred to other nutrients or combinations of nutrients for use in food‐based rather than supplement‐based intervention trials, which may be more acceptable to women during pregnancy or possibly other population groups who have suboptimal micronutrient intake.

## Conflict of Interest

NH is employed by Baxters Food Group. JC, LC, HM, PD, and GD declare no conflicts of interest.
